# Research of the preferred style of coping stress in relation to locus of control among healthcare workers during the COVID-19 pandemic

**DOI:** 10.1192/j.eurpsy.2023.494

**Published:** 2023-07-19

**Authors:** D. Muric

**Affiliations:** Institute of oncology, Clinical center of Montenegro, Podgorica, Montenegro

## Abstract

**Introduction:**

The locus of control is associated with a variety of psychological concepts, theories and researches, including learned helplessness, which is explained in way that person has learned to act helpless even when they actually have control over their situation or the ability to change a circumstance or outcome. In this scientific work, the aim is to examine the corelation between the locus of control and stress coping strategies in a group of health care workers. A sample is 110 respondents, of different age, gender, ages and educational degrees.

**Objectives:**

In this research, the aim is to examine the connection between locus of control and coping strategies among healthcare workers during the Covid-19 epidemic. The research results showed that there are no statistically significant differences in the relationship between stress coping strategies and locus of control in relation to men and women and age. Also, there is a statistically significant difference in the stress coping strategy focused on avoidance in relation to the locus of control, i.e. it turned out that the coping strategy focused on avoidance is more pronounced in respondents with an internal locus of control than in those with an external locus of control.

**Methods:**

The research was conducted at the Clinical Center of Montenegro, in Podgorica, in March 2022, through an online program, due to the epidemiological situation. The sample consists of 110 respondents, of both sexes and aged from 20 to 65 years old, who were chosen by the method of random selection. The CISS and RI-E scales were used.

**Results:**

The research showed that there is a connection between the locus of control and strategies for overcoming stress, but that there are no statistically significant differences in the connection between strategies for overcoming stress and locus of control in relation to men and women and their age, as well as that there is a statistically significant difference in the strategy coping with stress focused on avoidance in relation to locus of control, i.e. it was shown that the coping strategy focused on avoidance is more pronounced in respondents with an internal locus of control than in those with an external locus of control.

**Image:**

**Figure:**
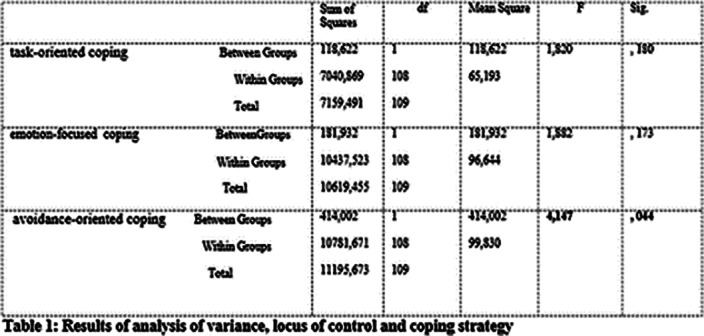

**Image 2:**

**Figure:**
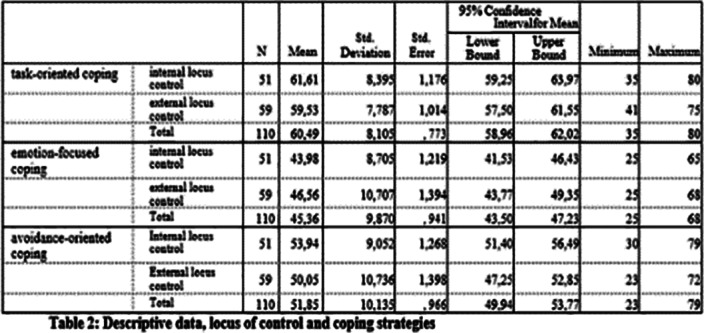

**Image 3:**

**Figure:**
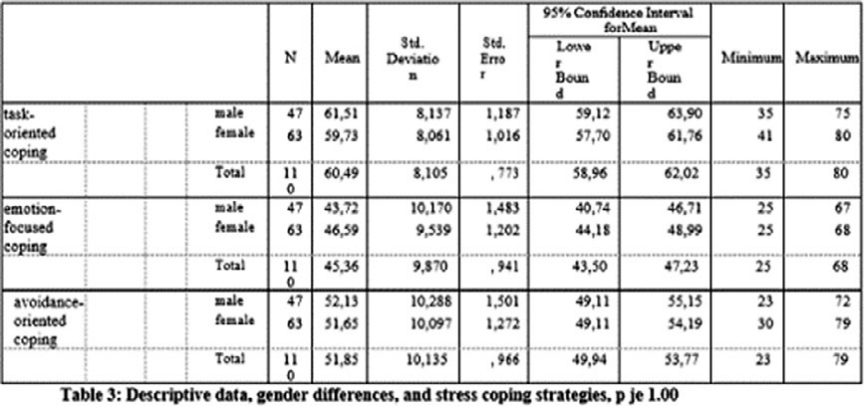

**Conclusions:**

This research actually confirmed the importance of individuality and various factors that can affect a person, and because of this, it was very likely that not all hypotheses could be answered in the way the author expected before the research began. With this, it can be assumed that the personality of a person can hardly be related to broad styles of coping with stress, and that generalizations regarding gender, age, education and work experience cannot be made, because there are predominantly individual differences in the development of an individual.

**Disclosure of Interest:**

None Declared

